# Early chronic suppression of microglial p38α in a model of Alzheimer’s disease does not significantly alter amyloid-associated neuropathology

**DOI:** 10.1371/journal.pone.0286495

**Published:** 2023-05-31

**Authors:** David J. Braun, Hilaree N. Frazier, Verda A. Davis, Meggie J. Coleman, Colin B. Rogers, Linda J. Van Eldik

**Affiliations:** 1 Sanders-Brown Center on Aging, University of Kentucky, Lexington, Kentucky, United States of America; 2 Department of Neuroscience, University of Kentucky, Lexington, Kentucky, United States of America; Torrey Pines Institute for Molecular Studies, UNITED STATES

## Abstract

The p38 alpha mitogen-activated protein kinase (p38α) is linked to both innate and adaptive immune responses and is under investigation as a target for drug development in the context of Alzheimer’s disease (AD) and other conditions with neuroinflammatory dysfunction. While preclinical data has shown that p38α inhibition can protect against AD-associated neuropathology, the underlying mechanisms are not fully elucidated. Inhibitors of p38α may provide benefit via modulation of microglial-associated neuroinflammatory responses that contribute to AD pathology. The present study tests this hypothesis by knocking out microglial p38α and assessing early-stage pathological changes. Conditional knockout of microglial p38α was accomplished in 5-month-old C57BL/6J wild-type and amyloidogenic AD model (APPswe/PS1dE9) mice using a tamoxifen-inducible Cre/loxP system under control of the *Cx3cr1* promoter. Beginning at 7.5 months of age, animals underwent behavioral assessment on the open field, followed by a later radial arm water maze test and collection of cortical and hippocampal tissues at 11 months. Additional endpoint measures included quantification of proinflammatory cytokines, assessment of amyloid burden and plaque deposition, and characterization of microglia-plaque dynamics. Loss of microglial p38α did not alter behavioral outcomes, proinflammatory cytokine levels, or overall amyloid plaque burden. However, this manipulation did significantly increase hippocampal levels of soluble Aβ42 and reduce colocalization of Iba1 and 6E10 in a subset of microglia in close proximity to plaques. The data presented here suggest that rather than reducing inflammation *per se*, the net effect of microglial p38α inhibition in the context of early AD-type amyloid pathology is a subtle alteration of microglia-plaque interactions. Encouragingly from a therapeutic standpoint, these data suggest no detrimental effect of even substantial decreases in microglial p38α in this context. Additionally, these results support future investigations of microglial p38α signaling at different stages of disease, as well as its relationship to phagocytic processes in this particular cell-type.

## Introduction

The mitogen activated protein kinase (MAPK) p38 has been found to be involved in a wide array of cellular responses throughout the body and brain, but its role in neuroinflammation is a canonical process, particularly in microglia [1–3]. In the context of Alzheimer’s disease (AD), amyloid beta (Aβ) contributes to p38-mediated release of proinflammatory cytokines both directly via increasing kinase activity and phosphorylation [4–9], as well as indirectly by promoting microglial activation and production of reactive oxygen species (ROS) [5, 7, 10–16]. In turn, the release of proinflammatory cytokines from microglia activates p38 in other cell-types which further exacerbates the inflammatory phenotype [17]. Thus, reducing p38 signaling in the CNS may represent a relevant therapeutic approach for the treatment of neuroinflammation in AD and other neurodegenerative diseases. Indeed, this hypothesis is well-supported in the literature [18, 19], and our lab has reported a beneficial effect of p38 inhibition in primary microglia *in vitro* [20] and in studies of neuroinflammatory insult *in vivo* [21–23]. Importantly, these changes were also associated with a rescue of behavioral deficits. We observed similar beneficial effects in an animal model of TBI, where microglia-specific genetic knock-out (KO) of p38α reduced levels of IL-6, IL-1β, and TNFα and decreased recruitment of inflammatory monocytes in injured mice [24]. Taken together, these data indicate that a primary mechanism of benefit of p38 inhibition in the context of neuroinflammatory damage may be the reduction of microglial neuroinflammatory responses.

Pharmacologic inhibitors of p38 are under active clinical investigation as treatments for AD by our group and others (e.g. neflamapimod). To maximize the odds of clinical success for this class of therapeutic intervention, we are in the process of defining cell-specific effects of p38α inhibition in the AD context. Microglial neuroinflammatory responses in AD are multifaceted, with some ameliorating and some exacerbating AD-associated pathology in a complex temporal manner [25]. Given advances in defining amyloid-positive but cognitively normal participants for AD trials (pre-clinical AD), and the success of this approach for early intervention in the lecanemab trials [26–28], we chose to selectively suppress microglial expression of p38α in an animal model of early-stage AD (i.e., detectable brain amyloidosis prior to the onset of memory impairments). To accomplish this, we generated WT and APP/PS1 AD model mice deficient in p38α using an inducible, microglia-specific Cre/loxP system. Microglial p38α was suppressed in adult animals subsequent to the age of first plaque deposition in this model. Animals were then assessed for behavioral performance using the open field (OF) and radial arm water maze (RAWM) tests, followed by microglial RNAseq analysis, measures of amyloid burden and proinflammatory cytokine levels, and characterization of microglia-plaque associations in the hippocampus. We report here that early and chronic loss of microglial p38α did not detrimentally affect behavioral performance, nor alter cytokine levels or overall plaque numbers. Interestingly, p38α ablation did significantly increase soluble Aβ42 and average plaque size, as well as decrease the volume of Aβ contained within plaque-associated microglia. These results are consistent with a role for p38α in mediating microglia-plaque interactions, but also indicate that even a substantial loss of microglial p38α signaling does not significantly impair protective microglial functions in this model of early disease.

## Materials and methods

### Animals and experimental design

**Fig 1:**
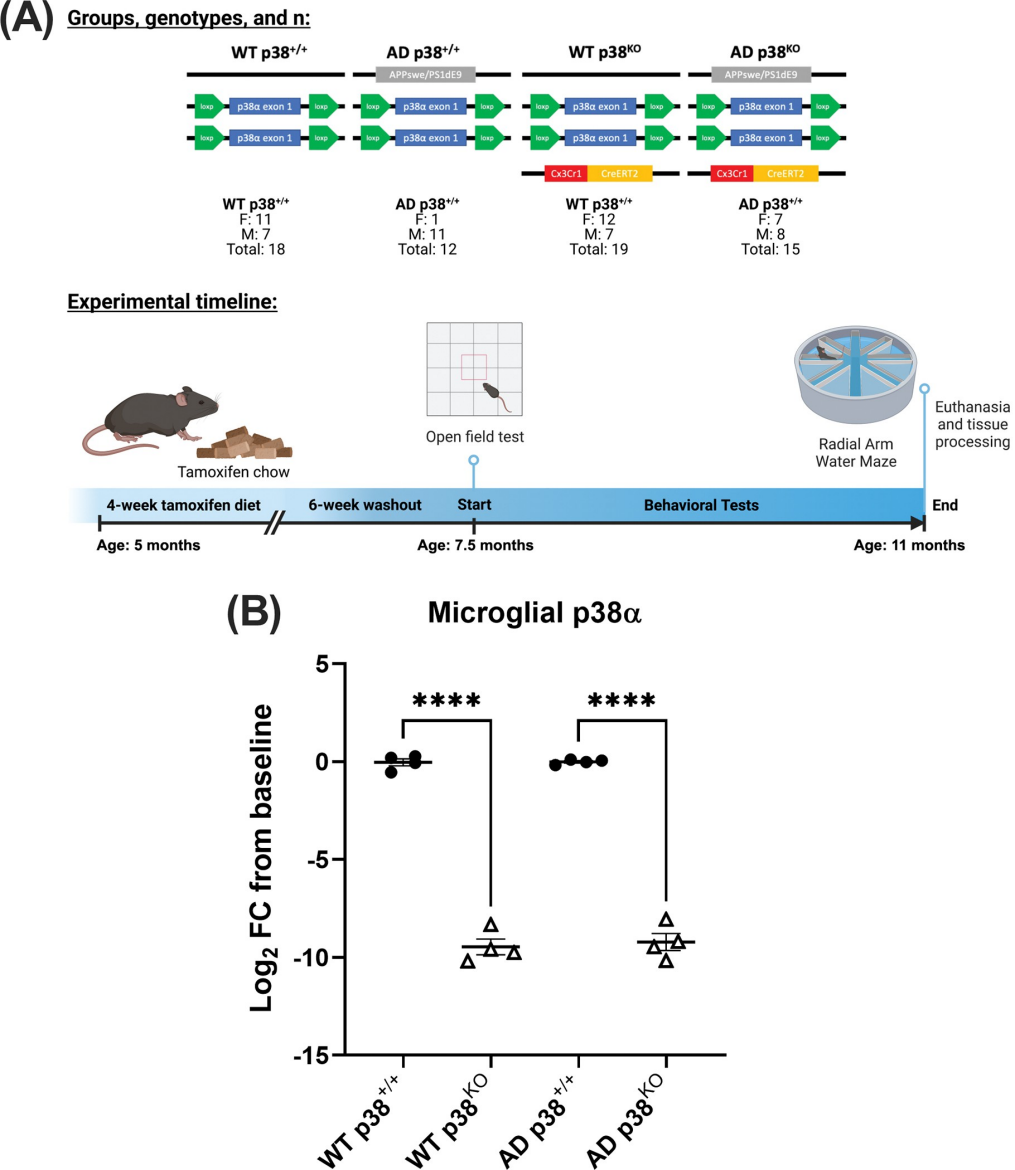
Experimental design and model validation. (A) Diagram of overall study design. Wild-type (WT) and APPswe/PS1dE9 (AD) mice were bred to carry p38α exon 1 flanked by loxP sites and a tamoxifen-inducible Cre recombinase gene under control of a *Cx3Cr1* promoter. At 5 months of age, animals were fed a tamoxifen diet for 4 weeks, then transferred back to normal chow to allow for replenishment of p38α-expressing peripheral myeloid cells. Behavioral testing began at 7.5 months of age for OF measures and 11 months of age for RAWM measures. Behavioral assessment was followed by euthanasia and tissue processing for additional endpoint measures. (B) RNAseq of isolated microglia from WT p38^+/+^, WT p38^KO^, AD p38^+/+^, and AD p38^KO^ mice (n = 4 per group). Animals from both WT and AD p38^KO^ groups had significant decreases in p38α transcript levels (>8 log_2_ fold change [FC]) compared to their respective p38^+/+^ control groups (1-way ANOVA; *F*_(3,12)_ = 297.4, *p* ≤ 0.0001), indicating that the genetic manipulation was successful. ****p ≤ 0.0001.

The APPswe/PS1dE9 mouse model of AD [29] (RRID:MMRRC_034832-JAX) was bred to mice carrying p38α exon 1 flanked by loxP sites (p38^fl/fl^) [30] and a tamoxifen-inducible Cre recombinase gene under control of the *Cx3cr1* promoter (Cx3cr1-creERT2^+/-^) [31] (RRID:IMSR_JAX:020940) on a congenic C57BL/6J background. At 5 months of age, all mice were switched from standard rodent chow (Envigo, #TD.2918) to food containing 400 ppm tamoxifen (Envigo, #TD.130860) for 4 weeks in order to generate wildtype (WT) and AD-type mice with normal microglial p38 expression (p38^+/+^) or with knockout of microglial p38 (p38^KO^). The four groups and underlying genotypes are referred to as follows: WT p38^+/+^ (APPswe/PS1dE9^-/-^, p38^fl/fl^, Cx3cr1-creERT2^-/-^), WT p38^KO^ (APPswe/PS1dE9^-/-^, p38^fl/fl^, Cx3cr1-creERT2^+/-^), AD p38^+/+^ (APPswe/PS1dE9^+/-^, p38^fl/fl^, Cx3cr1-creERT2^-/-^), and AD p38^KO^ (APPswe/PS1dE9^+/-^, p38^fl/fl^, Cx3cr1-creERT2^+/-^) (Fig 1A). To allow for turnover of peripheral CX3CR1-expressing immune cells, mice were then returned to the normal chow for 6–7 weeks, as prior work from our lab using a similar microglial p38αKO model reported that a 28-day washout period was sufficient to allow turnover of >96% of peripheral (Cd11b-) myeloid cells [24]. At ~7.5 months of age, mice underwent behavior testing beginning with the OF assay to test for gross motor/behavioral abnormalities that might interfere with later behavioral testing. After completion of testing, mice were allowed to age until ~11 months of age before undergoing RAWM assessment, which was immediately followed by euthanasia for endpoint analyses. Mice were deeply anesthetized with 5% isoflurane and humanely euthanized by exsanguination.

Throughout the study, mice were housed 1–5 animals per cage (503.22 usable cm^2^), in a room maintained at 23°C and on a 14:10 h light/dark cycle with *ad libitum* access to food and water. Enrichment included 5x5 nestlet (Ancare, Bellmore, NY, USA), paper shredding, and a Backless Shack mouse house (Shepherd Specialty Papers, Amherst, MA). Prior to behavioral testing, mice were acclimated to investigators via tunnel handling using polycarbonate tunnels. To allow for behavioral testing in the dark phase, animals were transferred to reverse light cycle housing after the resumption of normal chow [32]. All animal experiments were conducted in compliance with the University of Kentucky institutional licensing committee for the care and use of animals (Institutional Animal Care and Use Committee—IACUC).

### Spontaneous open field (OF) and radial arm water maze (RAWM) behavioral testing

Mice (n = 12–18 per group) were first assessed for spontaneous OF, modified from [33]. Briefly, four identical square arenas (40 x 40 x 40 cm) were positioned under the ceiling mounted camera for simultaneous recording and tracking via ANY-maze software (v6.35; Stoelting Co., Wood Dale, IL). After one hour of testing-room habituation, mice were placed in the center of individual open-field chambers to freely explore the arena without interruption for 15 min. The tracking software virtually divided each arena into 16 equal squares measuring 10 x 10 cm^2^; the 4 center squares were considered “center” and the outer ring of squares were designated “perimeter.” The software recorded the total distance traveled by each mouse, their average speed, and the percent of time spent in the arena center. The arenas were cleaned with 70% ethanol and dried after each trial.

Three months following completion of testing, mice were assessed in an 8-arm RAWM as described in Alamed, Wilcock [34]. Briefly, the water maze consisted of a circular tank (1.2 m diameter) with stainless steel inserts to create 8 arms radiating from the center of the pool. An escape platform was located at the distal end of one arm (goal arm) just below the surface of the water. The escape platform was flagged with a removable laminate cue for the visible trials. Water was made opaque with nontoxic white paint. Three laminated black and white cues were equally spaced and affixed to the edge of the tank to provide distal visual (spatial) cues. The first day of the 2-day protocol consisted of alternating visible and hidden platforms over 12 trials, temporally spaced by placing mice into cohorts of 4. The final 3 trials on day 1 and all 15 trials on day 2 used a hidden escape platform to force mice to use a spatial strategy to identify the goal arm location. ANY-maze tracking software recorded the average speed, average distance, and number of errors made by each mouse. Errors were operationally defined as any time the animal entered an arm that was not the goal arm, as well as any time the mouse remained in a single zone for 15 s or more.

### Flow cytometry and RNAseq

A randomly selected subset of 6 mice per genotype was processed for microglial isolation. After transcardial perfusion with ~15 mL of 1x DPBS (Corning Life Sciences, Tewksbury, MA), the brain was removed and the hemispheres divided. The right hemisphere was post-fixed in 4% paraformaldehyde (PFA) for 24 h, cryopreserved with 30% sucrose, sectioned and frozen for immunohistochemical analyses (see below). The left hemisphere had olfactory bulb, brainstem, and cerebellum removed before being dounce-homogenized. A microglial-containing fraction of cells was separated by 30% Percoll gradient (GE, #17-5445-01) in RPMI 1640 (Gibco, #11835030). Cells were incubated with 1:100 Zombie NIR dye solution (BioLegend, #423106), and 1:50 CD11b-VioB (Miltenyi Biotec, #130-113-810, RRID:AB_2726327) and P2ry12-PE (BioLegend, #848004; RRID: AB_2721645) antibodies. Approximately 10,000 live cells co-expressing CD11b and P2ry12 were sorted per mouse using a Sony Cell Sorter and immediately lysed in RLT Plus buffer and RNA extracted by RNeasy Plus Micro Kit (Qiagen, #74034) according to manufacturer’s instructions. RIN scores and total RNA were measured with a Bioanalyzer at the University of Kentucky Health Care Genomics Core Laboratory. To increase the reliability of our measurements, we selected the top 4 samples per group with the highest RIN scores (M = 8.3, minimum = 6.7, maximum = 9.1). Samples were then sent to Novogene for transcriptome sequencing: cDNA library was generated with NEBNext Ultra II RNA kit and run on Illumina Platform PE150. Raw reads were aligned with STAR, quantified to mm10 –Ensembl Transcripts release 100. Transcripts were analyzed for differential expression with DESeq2 with default settings. Partek Flow (v10.0.22.0828; Partek Inc., Chesterfield, MO) was used for all statistical comparisons and identification of relevant signaling pathways for all transcripts measured here. All RNAseq data discussed in this publication have been deposited in NCBI’s Gene Expression Omnibus (Edgar et al., 2002) and are accessible through GEO Series accession #GSE222257.

### Meso Scale Discovery (MSD) ELISA of proinflammatory cytokines and soluble Aβ

The mice not reserved for microglial isolation and RNAseq were transcardially perfused with 50 ml 1X PBS (Cellgro Mediatech, #46-013-CM) and brain tissue taken as previously described (34). Briefly, the brain hemispheres were separated by midline bisection and the right hemisphere immediately post-fixed in 4% PFA for ~24 h and cryopreserved in 30% sucrose at 4°C. For MSD ELISA assessment of amyloid (n = 6–9 mice per group) and proinflammatory cytokines (n = 6–13 mice per group), the left hemisphere was dissected to remove hippocampus and overlying cortex, and these brain regions were homogenized in 1X PBS lysis buffer (1:20 w/v) containing 100X Halt Protease Inhibitor Cocktail with 0.5 M EDTA (Pierce, #78442) and 200 mM PMSF using a Bead Ruptor 24 homogenizer (Omni International Inc., Kennesaw, GA). Homogenates were centrifuged (12000xg, 20 min, 4°C) and supernatants collected for cytokine and Aβ measurements using V-PLEX Proinflammatory Panel 1 Mouse Kit (MSD, #K15048D) and V-PLEX Aβ Peptide Panel 1 (6E10) Kit (MSD, #K15200E), respectively. Two of 10 cytokines with >20% non-detects across the experiment were excluded from analysis (IL-12p70 and IL-4) leaving 8 successfully quantified: IL-10, IL-1β, IL-2, IL-5, IL-6, TNFα, and KC/GRO. All cytokine and Aβ peptide levels were calculated using Discovery Workbench software (v4.0; Meso Scale Diagnostics LLC, Rockville, MD) and normalized to total mg of the PBS-soluble protein fraction loaded per sample as determined by BCA assay (Thermo Fisher Scientific, #23225).

### Immunohistochemistry and immunofluorescence

Fixed samples were sectioned at 30 μm on a sliding cryotome and stored in cryoprotectant at -20°C until immunostained as previously described [35]. For immunohistochemical analyses, free-floating sections through the hippocampus were selected from each mouse (n = 9–12 mice per group, 6–8 sections per animal), then blocked (10% goat serum, 0.2% TX-100 in PBS) and incubated with 1:3000 biotinylated mouse anti-Aβ 6E10 (BioLegend, # 803007, RRID:AB 2564657). All sections underwent signal amplification with an ABC kit (Vectashield, #PK-4000) according to manufacturer’s instructions and were developed with 0.05% (w/v) 3,3’-diaminobenzidine tetrahydrochloride hydrate (MilliporeSigma, #D5637). Slides were imaged on an Aperio ScanScope XT digital slide scanner using a 20x magnification. For each section, the cortex and hippocampus were manually outlined by a blinded investigator using HALO software (v3.5; Indica Labs, Albuquerque, NM). Quantification of plaque number and size in AD mice was accomplished using the HALO Object Colocalization v2.1.5 algorithm. Minimum intensity thresholds were set using no-primary antibody control sections.

For immunofluorescent analyses, tissue sections from AD mice (n = 8–10 mice per group, 4–10 sections per animal) were selected and blocked as above, then incubated overnight in 1:2000 rabbit anti-Iba1 (FUJIFILM Wako Shibayagi, #019–19741, RRID:AB_839504), 1:800 Alexa647-conjugated mouse anti-Aβ 6E10 (BioLegend, #803021, RRID:AB_2783374), and 1:1000 goat anti-rabbit Alexa594 secondary (Thermo Fisher Scientific, #A-11012, RRID:AB_2534079). Sections were subsequently incubated for 5 min in 1% Thioflavin S solution (MilliporeSigma, #T1892) and coverslipped in ProLong Gold Antifade with DAPI (Thermo Fisher Scientific, #P36931). Slides were imaged for on a Nikon BioPipeline Slide scanner (Nikon, Tokyo, Japan) using a Nikon 10x Plan Apo lambda objective. Images were stitched together and processed through denoise.ai and clarify.ai software using NIS-Elements (v5.4, Nikon), then imported into HALO for assessment of microglia and plaques. Hippocampal regions were first manually outlined by a blinded investigator, then analyzed using the Object Colocalization FL v.2.1.4 algorithm in order to identify individual microglia (Iba1 [TxRed] positive objects) and plaques (double-positive Thioflavin S [FITC] and 6E10 [Cy5] objects). To exclude erroneous objects corresponding to debris and/or imaging artifacts, only Iba1+ objects between 45–4000 μm^2^ in area were considered microglia and only 6E10-Thioflavin S double-positive objects between 20–1400 μm^2^ in area were considered plaques.

### Analysis of confocal immunofluorescent images

To further characterize microglia-plaque dynamics, a subset of fluorescently stained sections containing hippocampal regions of similar area (180–300 cm^2^) were randomly selected using a number generator (n = 10 mice per group, 1 section per animal). For each section, 20 hippocampal plaques double-stained for 6E10 and Thioflavin S were randomly selected for high-resolution imaging on a Nikon A1R-HD confocal microscope. Z-stacked images across 18 μm (step size of 0.5 μm) were taken at 1024x1024 resolution using a Nikon Plan Apo lambda 40x objective. A 2x optical zoom was applied to increase magnification to 80x during imaging. Images centered on each plaque of interest were first post-processed in NIS-Elements using denoise.ai software and the Richardson-Lucy deconvolution method, then imported into Imaris software for analysis (v9.7; Oxford Instruments, Abingdon, United Kingdom). Individual objects positive for Iba1, Thioflavin S, and 6E10 were automatically detected using Imaris surface algorithms that were thresholded for min-max signal intensity (TxRed: 390–2429, FITC: 647–4255, Cy5: 437–48245) and object size (TxRed: >4107 voxels, FITC: >10 voxels, Cy5: >10 voxels). Any image that included additional plaques residing within 50 μm of the central plaque of interest was removed from the analysis, as this would confound attempts to measure microglia associated with only the central plaque. Measures of microglial number and co-localization of Iba1 and 6E10 positive objects were then obtained for each image (n = 4–9 plaques per animal).

### Statistics and figure generation

Unless otherwise noted, all statistics were performed with Prism v9.4.1 (GraphPad Software, San Diego, CA). Graphs show group means with error bars representing standard error of the mean (SEM). Significance levels were set at α = 0.05. The minimal dataset for all analyses is attached as S1 Table.

## Results

### Microglial p38α loss does not impair behavioral performance of early-stage AD model mice

**Fig 2:**
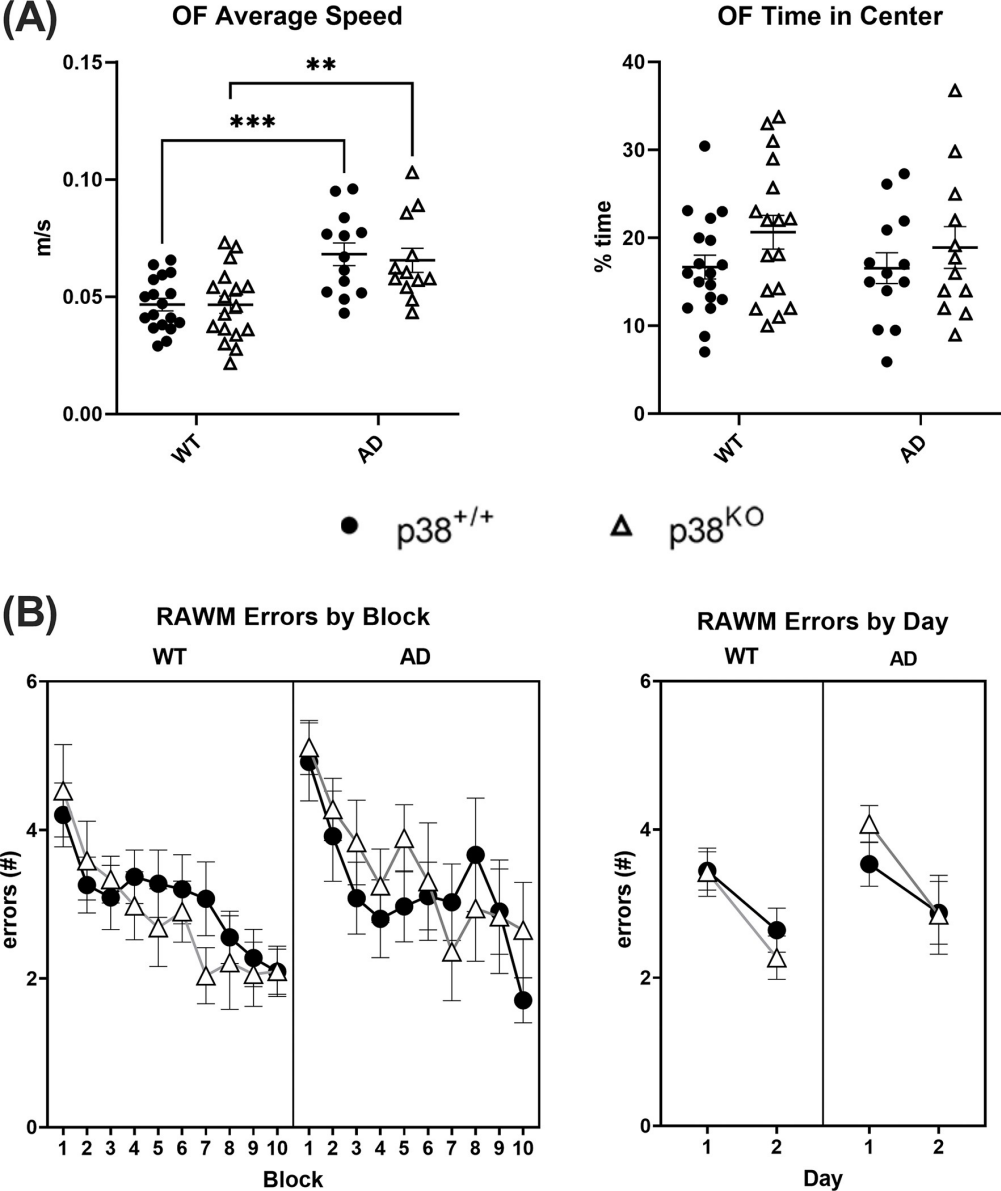
Behavioral assessment of WT and AD model mice. (A) Results of open-field (OF) testing in WT and AD animals (n = 12–18 per group). A main effect of genotype was detected on measures of speed (m/s), suggesting a hyperlocomotive phenotype in the AD animals (2-way ANOVA; *F*_(1,56)_ = 25.38, *p* ≤ 0.0001). This was unchanged in response to p38α KO (*p* ≥ 0.05). Time spent in the center of the field (%) was not altered by either genotype or p38α status (2-way ANOVA; *p* ≥ 0.05). Asterisks represent Šídák’s multiple comparisons pos-hoc tests (**p ≤ 0.01; ***p ≤ 0.001). (B) Results of radial arm water maze (RAWM) testing visualized across blocks (3 consecutive trials each) or averaged across days (n = 12–18 per group). All groups showed an improvement in performance over time indicated by a significant reduction in the # of errors (2-way ANOVA; *F*_(1,56)_ = 25.38, *p* ≤ 0.0001), although no effect of either genotype or p38α status was detected (*p* ≥ 0.05, respectively).

To confirm the efficacy of tamoxifen administration and loss of p38α, Cd11b+/P2ry12+ microglial cells from a subset of mice (n = 4 per group) were sorted and isolated for RNAseq analysis (Fig 1B). Similar substantial reductions in the major p38α transcript (encoding isoform 1) were found in both WT and AD p38^KO^ mice relative to their respective p38^+/+^ control groups (1-way ANOVA; *F*_(3,12)_ = 297.4, *p* < 0.0001), indicating the manipulation was successful. Six weeks after completing tamoxifen diet, all mice began longitudinal behavioral testing around 7.5 months of age beginning with the OF assay (Fig 2A). The APPswe/PS1dE9 mice exhibited a hyperlocomotive phenotype relative to WT littermates (2-way ANOVA; *F*_(1,56)_ = 25.38, *p* < 0.0001), but this was not altered by loss of microglial p38α (*p* > 0.05). There were no differences in % time in center between AD and WT mice, nor between p38α^+/+^ and p38^KO^ groups, at this age (2-way ANOVA; *p* > 0.05). Following completion of testing, mice were allowed to continue aging for 3 additional months before undergoing assessment in the 8-arm RAWM in an attempt to discern whether p38α loss might alter spatial learning and memory (Fig 2). All four groups improved in performance as assessed by a decrease in the number of incorrect arm entries (errors) over time (2-way ANOVA; *F*_(1,56)_ = 25.38, *p* < 0.0001). However, there was no significant effect of either genotype or p38α suppression across any of the groups (Fig 2B), indicating that even a substantial inhibition of microglial p38 in early disease stages does not worsen function.

### Microglial p38α loss does not alter the disease-associated microglia (DAM) transcriptional signature nor alter proinflammatory cytokines

**Fig 3:**
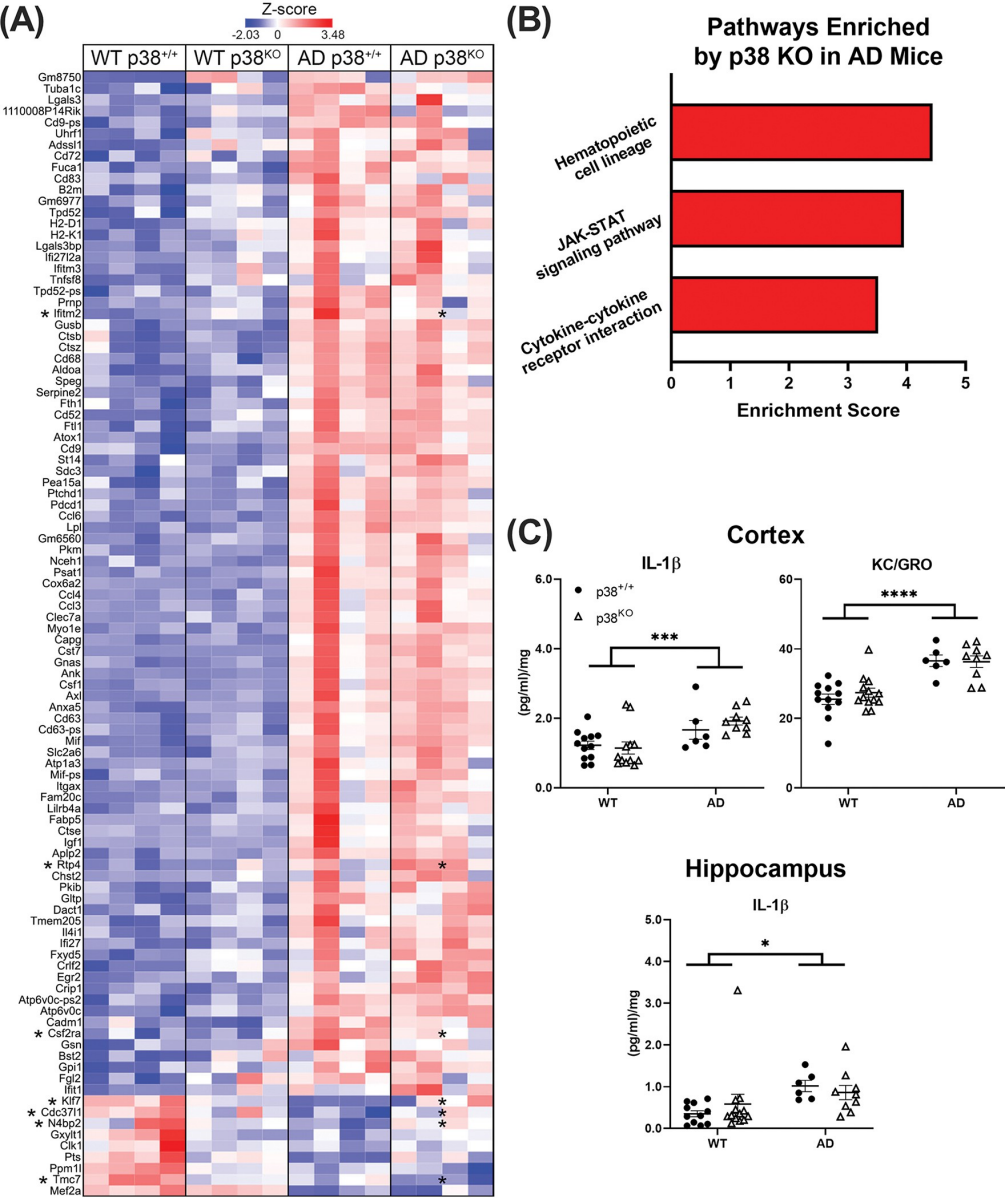
RNAseq analysis and MSD ELISA of proinflammatory cytokines in WT and AD mice. (A) RNAseq data obtained from isolated microglia (n = 4 mice/group). Analysis of more than 3,000 genes revealed 100 that were affected by genotype (DESeq2 with default settings; FDR corrected *p* ≤ 0.05). Of these, 7 were significantly altered by p38α suppression in the AD group (*Ifitm2*, *Rtp4*, *Csf2ra*, *Klf7*, *Cdc37l1*, *N4bp2*, and *Tmc7*). Significance between AD p38^+/+^ and AD p38^KO^ is indicated by asterisks (*). (B) Further analysis showed that KO of microglial p38α in AD mice was associated with a significant enrichment in pathways mediating hematopoietic stem cell development, JAK-STAT signaling, and cytokine-cytokine receptor interactions (KEGG pathway analysis; *p* ≤ 0.05). (C) Levels of the proinflammatory cytokines IFNγ, IL-10, IL-1β, IL-2, IL-5, IL-6, KC/GRO, and TNFα were measured in the cortex and hippocampus of WT and AD mice (n = 6–13 per group). Only IL-1β and KC/GRO were significantly altered by genotype, and the latter only in the cortex (2-way ANOVAs; IL-1β cortex–*F*_(1,35)_ = 12.93, *p* = 0.001, hippocampus–*F*_(1,35)_ = 5.99, *p* = 0.020; KC/GRO cortex–*F*_(1,36)_ = 38.79, *p* ≤ 0.0001). Microglial KO of p38α did not affect any of the cytokines tested here (*p* ≥ 0.05). Data represent means ± SEM. *p ≤ 0.05; ***p ≤ 0.001; ****p ≤ 0.0001.

Microglia were isolated by flow cytometry and subjected to RNAseq analysis. Representative flow cytometry plots are shown in S1 Fig. As expected, analysis of RNAseq data from isolated microglia (n = 4 mice per group) showed that genomic profiles differed significantly between WT and AD animals (Fig 3A), although little to no effect of p38α KO on gene signatures associated with microglial activation or the DAM phenotype were detected. However, compared to AD p38^+/+^ mice, AD p38^KO^ animals had significant alterations in the expression of 7 genes, many of which are known to be involved with pathways relevant to AD-pathology (Fig 3B). Specifically, these genes have been associated with the regulation of immune responses and immune cell development (*Ifitm2*, *Csf2ra*, *Rtp4*) [36–38], tau folding and heat shock protein processes (*Cdc37l1*) [39, 40], and maintenance of neuronal function in the hippocampus (*Klf7*) [41]. Compared to WT animals, levels of *Klf7*, *Cdc37l1*, *Rtp4*, and *N4bp2* were upregulated in the AD p38^+/+^ group, while *Ifitm2*, *Tmc7*, and *Csf2ra* were reduced. Suppression of p38α in AD mice shifted the expression of 5 of these genes (*Iifitm2*, *Csf2ra*, *Klf7*, *Cdc37l1*, and *N4bp2*) to levels similar to those seen in WT animals, but enhanced the AD-associated changes in *Rtp4* and *Tmc7*, suggesting differential effects of p38α inhibition across genes. To assess the impact of these transcriptional changes on the overall neuroinflammatory milieu, levels of the proinflammatory cytokines IFNγ, IL-10, IL-1β, IL-2, IL-5, IL-6, KC/GRO, and TNFα were measured in dissected hippocampus and overlying cortical regions from WT and AD mice (n = 6–13 per group). In the cortex, IL-1β and KC/GRO were found to be significantly elevated by genotype (2-way ANOVA; *F*_(1,35)_ = 12.93, *p* = 0.001 and *F*_(1,36)_ = 38.79, *p* < 0.0001, respectively), while in the hippocampus, only IL-1β was increased (*F*_(1,35)_ = 5.99, *p* = 0.020) (Fig 3C). Suppression of microglial p38α had no effect on any of the cytokines tested (*p* > 0.05).

### Suppression of microglial p38α alters aspects of amyloid deposition in the hippocampus

**Fig 4:**
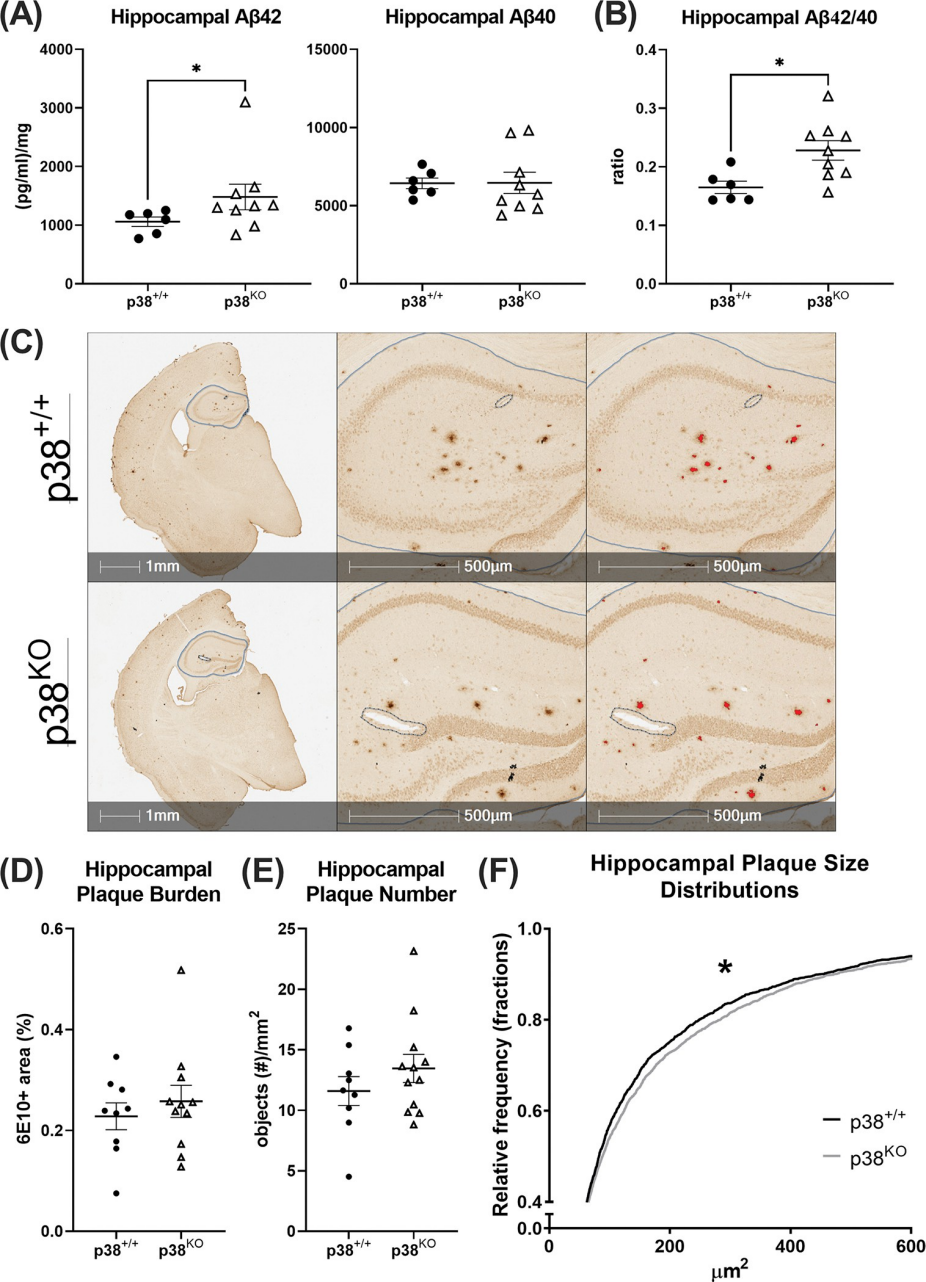
Amyloid-related measures in AD mice using MSD ELISA and immunohistochemical techniques. (A, B) The hippocampus was isolated from the left hemisphere of AD mice and homogenized for assessment of soluble Aβ40 and Aβ42 using MSD ELISA (n = 6–9 per group). While Aβ40 levels did not differ between groups (Student’s *t*-Tests; *p* ≥ 0.05), both soluble Aβ42 (Mann-Whitney *U* Test; *p* = 0.036) and Aβ42/40 ratios (Student’s *t*-test; *p* = 0.014) were significantly increased in the AD p38^KO^ group. (C) Representative image of tissue sections stained for amyloid (6E10) and analyzed using HALO imaging software (n = 9–11 per group). For each section, the hippocampus was manually outlined by a blinded investigator using HALO software (left panel, solid blue line). Regions of exclusion (dotted blue line) were drawn around debris, such as hair or fuzz, and around any torn areas of tissue in order to remove these regions from the analysis. The analysis algorithm was then manually thresholded for signal intensity and object size to ensure that only clear, distinct 6E10+ plaques were included (right panel, red overlays). (D-F) Objects were then quantified to obtain measures of hippocampal plaque burden (6E10+ % area), number (objects/mm^2^), and plaque size distributions (fractions). Analysis revealed a modest, but significant, effect of microglial p38α suppression on the size distribution of 6E10+ plaques between groups (Kolmogorov-Smirnov test; *p* = 0.049), with AD p38^KO^ mice having larger plaques (median = 88.22 μm^2^) compared to AD p38^+/+^ animals (median = 82.39 μm^2^). Measures of plaque burden and plaque number did not differ between groups (Student’s *t*-Test; *p* ≥ 0.05, respectively). Data represent means ± SEM. *p ≤ 0.05.

To characterize the impact of microglial p38α loss on amyloid deposition in AD mice (n = 6–9 per group), levels of soluble Aβ40 and Aβ42 ([pg/ml]/mg), Aβ42/40 ratios, overall plaque burden (6E10+ % area), and plaque numbers (6E10+ objects/mm^2^), were measured in cortical and hippocampal regions using MSD ELISA and immunohistochemistry (Fig 4). Consistent with our previous studies [21, 23], microglial p38α loss did not alter cortical levels of soluble Aβ42 (p38^+/+^ mean = 1777 ± 160.8 (pg/ml)/mg, p38^KO^ mean = 1948 ± 523.4 (pg/ml)/mg; Mann-Whitney *U* Test; *U*_(24)_, *p* ≥ 0.05) or Aβ40 (p38^+/+^ mean = 5818 ± 207.6 (pg/ml)/mg, p38^KO^ mean = 5547 ± 365.5 (pg/ml)/mg; Student’s *t*-Test; *t*_(0.56)_, *p* ≥ 0.05), nor cortical plaque burden (p38^+/+^ mean = 0.283 ± 0.031% area, p38^KO^ mean = 0.334 ± 0.025% area; Student’s *t*-Test; *t*_(1.30)_, *p* ≥ 0.05). However, in the hippocampus, a significant increase in soluble Aβ42 (Mann Whitney *U* Test; *p* = 0.036) and Aβ42/40 ratios (Student’s *t*-Test; *p* = 0.014) were detected in AD p38^KO^ animals compared to AD p38^+/+^, suggesting that suppression of p38α may alter some aspects of amyloid deposition in this region (Fig 4A and 4B). Analysis of overall plaque burden and plaque number in the hippocampus showed no change in response to p38α KO in this model (Student’s *t*-Tests; *p* > 0.05; Fig 4C–4E). However, to further probe potential alterations in plaque morphology, the plaque size frequency distributions were compared in AD animals [42]. Results revealed a significant, though modest, shift in the size distribution of 6E10+ plaques between groups (Kolmogorov-Smirnov test; *p* = 0.049), with AD p38^KO^ mice having larger plaques (median = 88.22 μm^2^) compared to AD p38^+/+^ animals (median = 82.39 μm^2^) (Fig 4F). Together, these data suggest a potential effect of p38α loss on microglial uptake or deposition of Aβ, which we assessed directly via confocal imaging.

### Suppression of microglial p38α significantly reduces amyloid colocalization to Iba1+ objects

**Fig 5:**
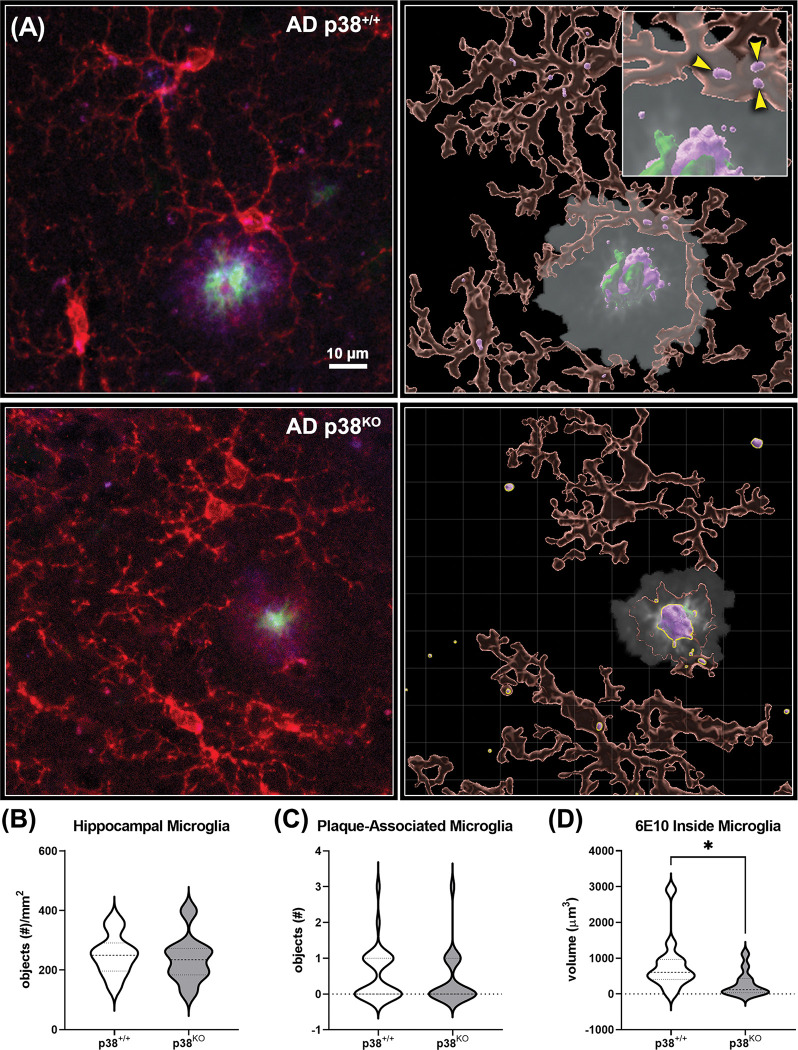
Microglia-plaque dynamics measured in the hippocampus of p38^+/+^ and p38^KO^ AD mice. (A) Left panels: Representative images of tissue sections probed for microglia (Iba1: TxRed [red]), fibrillar amyloid deposits (Thioflavin S: FITC [green]), and Aβ peptides (6E10: Cy5 [purple]) obtained on a Nikon confocal microscope. Z-stack images across 18 μm were obtained for each plaque of interest and post-processed using NIS-Elements software (n = 10 animals per group, 20 plaques per animal). Right panels: Images were imported into Imaris software, where individual surface objects were detected using built-in algorithms manually thresholded for signal intensity and object size. For analysis of plaque-associated microglia, a region of interest restricting measures to only those cells residing within 15 μm of the plaque was applied to each image (white semi-translucent region). Right inset: Close up of an Imaris reconstruction showing a plaque-associated microglial cell. Aβ peptides (purple objects) contained within the cell body are indicated by yellow arrows. (B) Prior to analysis of plaque-associated microglia, overall Iba1 positive objects in the hippocampus were obtained for each section (n = 8–10 per group). No change was detected between groups (Student’s *t*-Test; *p* ≥ 0.05). (C, D) The number of microglia residing within 15 μm of the plaques did not change in response to p38α suppression (Student’s *t*-Test; *p* ≥ 0.05). However, quantification of Aβ volume (μm^3^) in plaque-associated microglia revealed a significant decrease in AD p38^KO^ mice compared to p38^+/+^ animals (Student’s *t*-Test; *p* = 0.011), suggesting that p38α suppression may potentially affect microglia-plaque dynamics and/or phagocytic processes in this region. Data represent means ± SEM. *p ≤ 0.05.

The effect of p38α loss on microglial number and morphology was assessed in AD mice (n = 10 per group) using immunofluorescent techniques in combination with high-resolution confocal microscopy (Fig 5). Assessment of overall microglial numbers in the hippocampus (Iba1+ objects/mm^2^) showed no difference between p38^+/+^ and p38^KO^ groups (Student’s *t*-Test; *p* > 0.05; Fig 5B). To better characterize microglia-plaque dynamics, the analysis was then restricted to include only microglia residing within 15 μm [23] of their respective plaque (Fig 5A, inset). Analysis of these plaque-associated microglia showed no change in cell number following KO of p38α (Student’s *t*-Test; *p* > 0.05; Fig 5C). However, comparison of Iba1 and 6E10 colocalization in this subset of microglia revealed nearly a 2-fold reduction in 6E10+ amyloid volume (μm^3^) in the p38^KO^ group compared to p38^+/+^ animals (Student’s *t*-Test; *p* = 0.011), indicating a potential reduction in microglial uptake of Aβ (Fig 5D).

## Discussion

In this study, we tested the effects of early and chronic microglial p38α suppression on cognitive performance, neuroinflammatory processes, and amyloid pathology in WT and APP/PS1 AD model mice. Although AD mice exhibited the expected hyperlocomotive phenotype during OF testing, spatial memory performance was not significantly different between WT and AD p38^+/+^ groups at the age tested, indicating (a) that we are modeling a pre-clinical phase of the disease wherein overall cognitive function is unimpaired despite the presence of amyloid pathology [43], and (b) substantial loss of microglial p38α signaling is not detrimental to at least this type of spatial memory. We further report that this lack of effect on functional measures is mirrored by non-detectable or only subtle effects on neuropathological markers. Although p38α loss was associated with microglial transcriptional alterations, there was no change in overall proinflammatory protein levels, microglial number, or plaque numbers. Interestingly, however, this manipulation was associated with a modest but significant increase in amyloid burden in the hippocampus. Specifically, microglial KO of p38α was associated with a shift in the median plaque size as well as with elevated levels of soluble Aβ42 and Aβ42/40 ratios (Fig 4). While the reduction in soluble Aβ42 without a concurrent change in Aβ40 is somewhat surprising (Fig 4C), some evidence has shown that microglial clearance mechanisms and degradation rates differ slightly between these peptides [44]. Further, loss of microglial p38α in AD mice was associated with significant reductions in the amount of amyloid co-localized within Iba1-postive cells <15 μm from a plaque (Fig 5D), which might represent changes in amyloid clearance mechanisms in these cells [45].

Although an exploration of the specific mechanisms underlying these observations is beyond the scope of the present study, it is worth following up in future investigations. Indeed, work has shown that modulation of p38 and downstream signaling effectors may alter microglia-plaque interactions and/or amyloid clearance, albeit in varying ways [46, 47]. For example, in 5xFAD mice, p38 inhibition reduced Aβ deposits and increased microglial phagocytic receptors [48]. In human peripheral blood monocytes, inhibition of p38 activation ameliorated the effects of Aβ-induced oxidative stress and cytotoxicity [49]. Similarly, in microglial cell culture models (HMC3 and BV-2 cells), acute administration of the p38 inhibitor BIRB was associated with significant reductions in Aβ phagocytosis [4]. However, another study performed in BV2 cells reported opposing results, with p38 suppression leading to increased, rather than decreased, phagocytosis of Aβ [50]. In our lab, we recently reported that *in vivo* administration of the p38α-specific inhibitor MW150 to APP/PS1 mice did not alter overall amyloid burden or colocalization of 6E10 and Iba1-positive microglia in the cortex, suggesting no effect on amyloid phagocytosis, though this manipulation did significantly increase the number of microglia residing in close proximity (<15 μm) to plaques (plaque-associated microglia) [23]. This latter effect on microglial recruitment was also detected in a recent study performed in p38α-deficient APP/PS1 mice, which reported a similar increase in the number of microglia surrounding Aβ deposits along with a decrease in overall Aβ deposition in the hippocampus [51], further supporting the role of p38 signaling in mediating microglia-plaque dynamics. Given these previous findings alongside the more subtle effects found in our current model, it may be that the effects of microglial p38 modulation on amyloid burden only become apparent at more advanced stages of disease.

RNAseq analysis did not identify changes in any genes known to mediate phagocytic processes or amyloid clearance mechanisms in this cell-type (potentially due to a lack of enrichment for specifically plaque-associated microglia), although we did find an association between loss of microglial p38α and altered expression of a small number of genes in AD mice (Fig 3A and 3B). Specifically, p38^KO^ in AD mice resulted in changes in pathways associated with hematopoietic cell lineage, JAK-STAT signaling, and with cytokine-cytokine receptor interactions, even though no effect of microglial p38α KO was detected on proinflammatory cytokine levels (S2 Fig). Given prior evidence that inhibiting p38 can reduce proinflammatory cytokines [1], one might have expected to see similar p38-mediated changes in cytokine levels here. However, it may be that other cell types (e.g. astrocytes [52] or neurons [9, 17, 48, 53–60]) contribute to proinflammatory cytokine expression more profoundly than microglia, or that other microglial signaling pathways aside from p38 predominate in this more chronic and milder neuroinflammatory state. This could explain, for example, why the same manipulation was able to reduce cytokine levels in the context of the much larger and acute proinflammatory TBI model [24]. Although our pre-selected markers of neuroinflammation were unchanged, several other neurodegenerative changes, including increased oxidative stress, apoptosis, and impaired neurotransmission, are also known to be altered by this signaling pathway [9, 61–64], so it will be important to further explore these endpoints in future work. Additionally, evidence for the beneficial impact of p38 suppression is not limited to models of amyloid pathology. In P301S Tau-transgenic mice, neuron-specific deletion of p38α significantly reduced p-Tau expression and neurofibrillary tangles in the cerebellum [65], suggesting that microglial p38 may potentially be more relevant in the context of tangle-associated or the combination of plaque and tangle-associated AD neuropathology.

The present work provides important evidence that inhibition of microglial p38 is not detrimental to amyloid-associated neuropathology; however, there are several important caveats worth highlighting. First, the KO of p38α was generated using a *Cx3cr1* promoter that resulted in these animals having a *Cx3cr1* haploinsufficiency. As disruption of CX3CL1/CX3CR1 signaling pathways in microglia has been shown to impair microglial responses, modify the neuroinflammatory profile, and alter Aβ deposition [66], this haploinsufficiency could have confounded potential p38-mediated effects in our model. Further, while CX3CR1 expression is limited to myeloid-derived cells, it is not specific to microglia, suggesting that infiltrating p38αKO peripheral cells, such as macrophages, could have contributed to some of the effects reported here. This is unlikely, however, as we reverted all animals back to standard chow for 6–7 weeks after tamoxifen exposure in order to allow turnover of CX3CR1-expressing cells in the periphery (Fig 1A). It is also important to note that while this study did include both male and female animals, the APPswe/PSEN1dE9 (C57BL6) mouse strain has a propensity for developing seizures beginning at ~3 months of age [67], and this resulted in the deaths of several animals during the course of experiments: 4 female AD p38^+/+^, 1 male AD p38^+/+^, 1 female AD p38^KO^, and 1 male p38^KO^. Although this implies the KO might be protective for females of this strain, deaths were too low to determine this. This also left the final sex ratios skewed across groups and underpowered to assess sex differences. This is not only an important caveat to consider in the context of disease progression [68], but also with respect to microglial p38 signaling, as some evidence has reported significant differences between male and female animals on measures of p38-mediated myeloid responses to Aβ as well as on the microglial transcriptome [69]. Finally, as AD presents with multiple pathologies and not only amyloid deposition [70], investigations of p38α in models that also present with other relevant neurodegenerative changes, such as those modeling Tau-associated pathologies [71], should be considered. Finally, p38 is a highly conserved and vital cellular signaling pathway–it may be the case that in our chronic model, redundant MAPK isoforms, activities, or parallel pathways are able to partially compensate for the KO, thus reducing the apparent effects of our intervention. Future work would benefit from characterizing not only the transcriptional effect of the KO, but also the functional effect on downstream pathways.

In summary, our results are consistent with a role for microglial p38α in the modulation of amyloid plaque dynamics *in vivo*. They also indicate that even a substantial reduction of p38α levels in microglia does not significantly impair important microglial disease-restricting processes, consistent with what we published previously using MW150 in a different AD model. It remains to be seen if p38’s role in microglial-plaque interactions becomes more important with age or disease progression, or what the effects on tau-related pathology might be. Further, the relative contribution of diminished p38 signaling in other cell-types is an important component not yet fully defined. Future characterization of p38 pathways in other animal models and cell-types, as well as across age and sex, remains highly relevant in the context of AD and other neurodegenerative diseases.

## Supporting information

S1 Fig Isolation of microglia using flow cytometry. (A) Representative heatmaps of microglia isolated from the left hemisphere of a WT p38^+/+^ mouse. Top Row: Briefly, for each animal, cells were identified using measures of cell granularity (side scatter area [SSC-A]) and cell size (forward scatter area [FSC-A]). The total cell population was then gated to exclude doublets (forward scatter height [FSC-H] vs. FSC-A) and non-viable cells (PE-Cyanine7 [PE-Cy7-A] vs. FSC-A). Bottom Row: The population of single, live cells was then gated to isolate microglia. First, subpopulations of cells expressing Cd11b (brilliant violet 421 vs. FSC-A) or P2yr12 (PE-A vs. FSC-A) were identified. This was followed by gating for co-expression of both markers (brilliant violent 421 vs. PE-A). All fluorescent intensity thresholds were set using compensation beads incubated with appropriate antibodies. Co-expressing cells were defined as “Microglia,” and immediately sorted and lysed for extraction of RNA.
(PDF)

S2 Fig Measures of proinflammatory cytokine levels in WT and AD mice. The hippocampus and overlying cortical tissue were isolated from the left hemisphere of p38^+/+^ and p38^KO^ WT and AD mice (n = 6–13 per group), then homogenized for assessment of 8 proinflammatory cytokines (IFNγ, IL-10, IL-1β, IL-2, IL-5, IL-6, KC/GRO, and TNFα) using MSD ELISA techniques. (A) While analysis of cortical cytokine levels showed a significant elevation in IL-1β (2-way ANOVA; *F*_(1,35)_ = 12.93, *p* = 0.001) and KC/GRO (*F*_(1,36)_ = 38.79, *p* ≤ 0.0001) in AD animals compared to WT, no impact of p38α suppression was detected across any of the cytokines measured here (*p* ≥ 0.05). (B) Similarly, the AD genotype significantly increased IL-1β in the hippocampus (2-way ANOVA; *F*_(1,35)_ = 5.99, *p* = 0.020), but no effect of p38α KO on cytokine levels was detected in this region (*p* ≥ 0.05). *p ≤ 0.05; ***p ≤ 0.001; ****p ≤ 0.0001.
(PDF)

S1 Table Minimal dataset for all analyses. (XLSX)
