# Supplementary material for: Early chronic suppression of microglial p38α in a model of Alzheimer’s disease does not significantly alter amyloid-associated neuropathology
Source: PLoS One. 2023 May 31;18(5):e0286495. doi: 10.1371/journal.pone.0286495 (PMC10231773; doi:10.1371/journal.pone.0286495)
Supplement: S1 Fig — (A) Representative heatmaps of microglia isolated from the left hemisphere of a WT p38+/+ mouse. Top Row: Briefly, for each animal, cells were identified using measures of cell granularity (side scatter area [SSC-A]) and cell size (forward scatter area [FSC-A]). The total cell population was then gated to exclude doublets (forward scatter height [FSC-H] vs. FSC-A) and non-viable cells (PE-Cyanine7 [PE-Cy7-A] vs. FSC-A). Bottom Row: The population of single, live cells was then gated to isolate microglia. First, subpopulations of cells expressing Cd11b (brilliant violet 421 vs. FSC-A) or P2yr12 (PE-A vs. FSC-A) were identified. This was followed by gating for co-expression of both markers (brilliant violent 421 vs. PE-A). All fluorescent intensity thresholds were set using compensation beads incubated with appropriate antibodies. Co-expressing cells were defined as “Microglia,” and immediately sorted and lysed for extraction of RNA. (PDF) [file pone.0286495.s001.pdf]

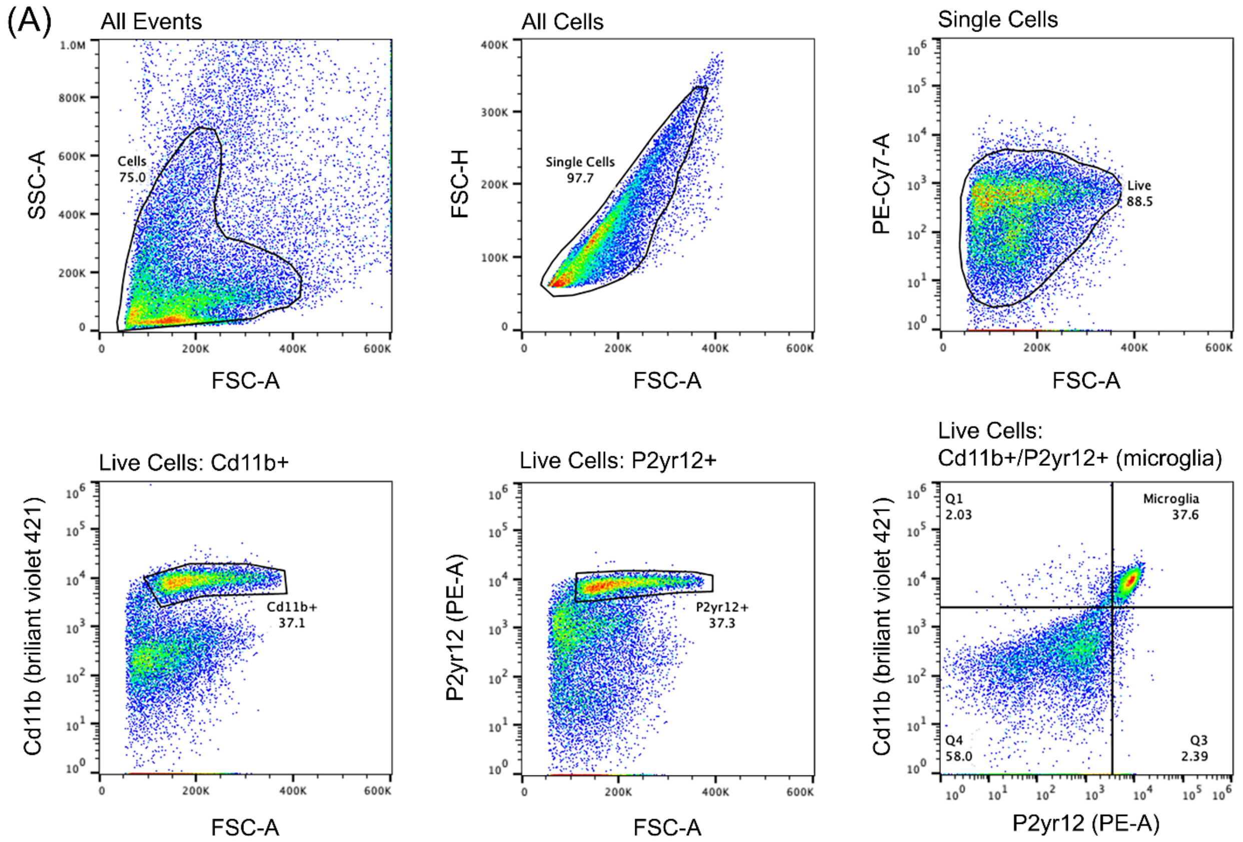

S1 Fig. Isolation of microglia using flow cytometry. (A) Representative heatmaps of microglia isolated from the left hemisphere of a WT p38$^{+/+}$ mouse. Top Row: Briefly, for each animal, cells were identified using measures of cell granularity (side scatter area [SSC-A]) and cell size (forward scatter area [FSC-A]). The total cell population was then gated to exclude doublets (forward scatter height [FSC-H] vs. FSC-A) and non-viable cells (PE-Cyanine7 [PE-Cy7-A] vs. FSC-A). Bottom Row: The population of single, live cells was then gated to isolate microglia. First, subpopulations of cells expressing Cd11b (brilliant violet 421 vs. FSC-A) or P2yr12 (PE-A vs. FSC-A) were identified. This was followed by gating for co-expression of both markers (brilliant violent 421 vs. PE-A). All fluorescent intensity thresholds were set using compensation beads incubated with appropriate antibodies. Co-expressing cells were defined as “Microglia,” and immediately sorted and lysed for extraction of RNA.
