# Supplementary material for: Early chronic suppression of microglial p38α in a model of Alzheimer’s disease does not significantly alter amyloid-associated neuropathology
Source: PLoS One. 2023 May 31;18(5):e0286495. doi: 10.1371/journal.pone.0286495 (PMC10231773; doi:10.1371/journal.pone.0286495)
Supplement: S2 Fig — The hippocampus and overlying cortical tissue were isolated from the left hemisphere of p38+/+ and p38KO WT and AD mice (n = 6–13 per group), then homogenized for assessment of 8 proinflammatory cytokines (IFNγ, IL-10, IL-1β, IL-2, IL-5, IL-6, KC/GRO, and TNFα) using MSD ELISA techniques. (A) While analysis of cortical cytokine levels showed a significant elevation in IL-1β (2-way ANOVA; F(1,35) = 12.93, p = 0.001) and KC/GRO (F(1,36) = 38.79, p ≤ 0.0001) in AD animals compared to WT, no impact of p38α suppression was detected across any of the cytokines measured here (p ≥ 0.05). (B) Similarly, the AD genotype significantly increased IL-1β in the hippocampus (2-way ANOVA; F(1,35) = 5.99, p = 0.020), but no effect of p38α KO on cytokine levels was detected in this region (p ≥ 0.05). *p ≤ 0.05; ***p ≤ 0.001; ****p ≤ 0.0001. (PDF) [file pone.0286495.s002.pdf]

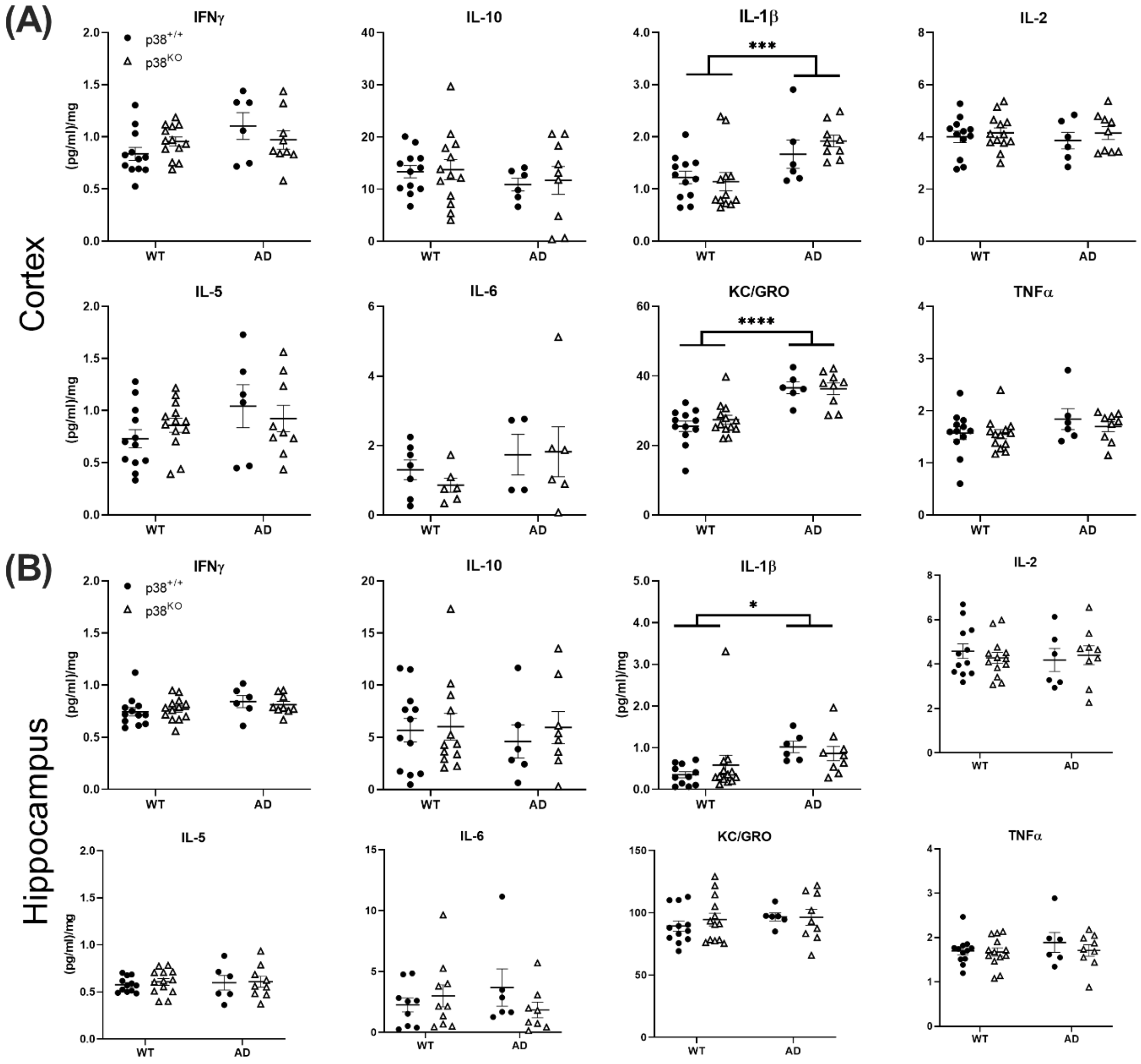

S2 Fig. Measures of proinflammatory cytokine levels in WT and AD mice. The hippocampus and overlying cortical tissue were isolated from the left hemisphere of $p38^{+/+}$ and $p38^{KO}$ WT and AD mice (n = 6-13 per group), then homogenized for assessment of 8 proinflammatory cytokines (IFNγ, IL-10, IL-1β, IL-2, IL-5, IL-6, KC/GRO, and TNFα) using MSD ELISA techniques. (A) While analysis of cortical cytokine levels showed a significant elevation in IL-1β (2-way ANOVA; $F_{(1,35)} = 12.93$, $p = 0.001$) and KC/GRO ($F_{(1,36)} = 38.79$, $p \leq 0.0001$) in AD animals compared to WT, no impact of p38α suppression was detected across any of the cytokines measured here ($p \geq 0.05$). (B) Similarly, the AD genotype significantly increased IL-1β in the hippocampus (2-way ANOVA; $F_{(1,35)} = 5.99$, $p = 0.020$), but no effect of p38α KO on cytokine levels was detected in this region ($p \geq 0.05$). *$p \leq 0.05$; ***$p \leq 0.001$; ****$p \leq 0.0001$.
